# The Role of Microbiota-Derived Vitamins in Immune Homeostasis and Enhancing Cancer Immunotherapy

**DOI:** 10.3390/cancers15041300

**Published:** 2023-02-18

**Authors:** Hasti Gholami, John A. Chmiel, Jeremy P. Burton, Saman Maleki Vareki

**Affiliations:** 1 Department of Pathology and Laboratory Medicine, Western University, London, ON N6A 3K7, Canada; 2 Department of Microbiology and Immunology, Western University, London, ON N6A 3K7, Canada; 3 Canadian Research and Development Centre for Probiotics, Lawson Research Health Research Institute, London, ON N6A 5W9, Canada; 4 Division of Urology, Department of Surgery, Western University, London, ON N6A 3K7, Canada; 5 London Regional Cancer Program, Lawson Health Research Institute, London, ON N6A 5W9, Canada; 6 Department of Oncology, Western University, London, ON N6A 3K7, Canada; 7 Department of Medical Biophysics, Western University, London, ON N6A 3K7, Canada

**Keywords:** vitamins, gut microbiome, immunotherapy, immune checkpoint inhibitors, fecal microbiota transplant

## Abstract

**Simple Summary:**

Despite clinical success, only a limited percentage of cancer patients are responsive to immunotherapy. Recently, gut microbiota modulation has been suggested as a tool to enhance immunotherapy efficacy, and mechanisms for these effects may be linked to microbial contributions—such as microbial-derived vitamins—to immune responses. While humans can acquire their vitamins from dietary sources, gut microbial-derived vitamins are crucial to the immune system’s function. The production of these vitamins can be altered by the bidirectional crosstalk between the immune system and the gut microbiome; however, their exact mechanism of action in bacterial communities and immune responses remains elusive. Further studies and clinical trials are needed to understand the role of microbial-derived vitamins in anti-tumor immune responses and their role in the efficacy of immunotherapies. This review will discuss the in-depth mechanisms of selective vitamins and their role in modulating immune responses, as well as their potential as immunotherapy enhancers.

**Abstract:**

Not all cancer patients who receive immunotherapy respond positively and emerging evidence suggests that the gut microbiota may be linked to treatment efficacy. Though mechanisms of microbial contributions to the immune response have been postulated, one likely function is the supply of basic co-factors to the host including selected vitamins. Bacteria, fungi, and plants can produce their own vitamins, whereas humans primarily obtain vitamins from exogenous sources, yet despite the significance of microbial-derived vitamins as crucial immune system modulators, the microbiota is an overlooked source of these nutrients in humans. Microbial-derived vitamins are often shared by gut bacteria, stabilizing bioenergetic pathways amongst microbial communities. Compositional changes in gut microbiota can affect metabolic pathways that alter immune function. Similarly, the immune system plays a pivotal role in maintaining the gut microbiota, which parenthetically affects vitamin biosynthesis. Here we elucidate the immune-interactive mechanisms underlying the effects of these microbially derived vitamins and how they can potentially enhance the activity of immunotherapies in cancer.

## 1. Introduction

Although microbes can be localized in different niches of the body, the human gastrointestinal (GI) tract is home to the largest communities of microorganisms, their products, and genomes—collectively known as the microbiome [1,2,3]. The gut microbiota comprises protozoa, viruses, and fungi, but bacteria encompass the largest biomass [1,4,5,6,7]. The homeostasis between the gut microbiome and immune system is important to overall human health [6,8]. The microbiota of the gut also has a wider systemic influence on the host as microbial metabolites and other components pass across intestinal barriers which influence distal organs and the immune system. Hence, the host’s immune system and the gut microbiota interact in a symbiotic relationship and collectively form a “superorganism” [9,10]. 

While microbial-derived vitamins have been covered less in the literature, extensive reviews have previously detailed the vast array of roles of the other gut-derived metabolites on immune function and immunotherapy response [11,12,13,14,15]. Vitamins are essential micronutrients that act as precursors for essential enzymes for vital biochemical reactions and cellular functions in the body [16,17,18]. Vitamins can modulate the host’s immune response in the gut, highlighting their role as critical mediators in the interplay between the gut microbiome and the immune response [19,20]. Additionally, some vitamins, such as vitamin C, can act as an effective adjuvant to immunotherapy, further supporting the immunomodulatory functions of vitamins [21,22,23,24]. Vitamin E has been shown to enhance the efficacy of immune checkpoint inhibitors (ICIs) in mouse models of breast cancer and melanoma [25]. Studies displayed significantly better objective response rates in patients with melanoma, breast, prostate, and kidney cancers who took vitamin E supplements with anti-programmed death-1 (PD-1) or anti-PD-1 ligand (PD-L1) inhibitors than those who did not [25]. These results hint at a potential role for using vitamins in promoting the efficacy of immunotherapy in various malignancies. 

Interestingly, gut bacteria produce specific vitamins alongside those obtained from dietary sources [26]. This review aims to elucidate the mechanisms underlying the effects of microbial-derived vitamins on immune response, the effect of deficiencies on immune-related diseases, and their potential effects on the response to immunotherapy against cancer. This review will discuss the roles of vitamins B6, B9, and B12 as an interconnected network of immunomodulators due to their similarities and overlapping functions in immune homeostasis. Furthermore, vitamin K2 will be analyzed alongside its interplay with vitamin D as a key inflammatory and immune response regulator. The many immunoregulatory roles of these vitamins have been simplified to provide an overall big-picture representation of their importance to the immune system and their potential promise in immunotherapeutic outcomes. 

## 2. Gut Microbiota and Immune Homeostasis 

The bidirectional crosstalk between the gut microbiota and the immune system is essential in developing and maintaining innate and adaptive immune responses. The host immune system impacts gut microbiota composition by properly balancing antigenic and infectious factors. Correspondingly, gut microbiota balance is critical to maintaining host immunity [7,27,28,29]. Specifically, the gut microbiota impacts the development of lymphoid tissues, sustains lymphocyte subpopulations in secondary immune organs, and helps local mucosal immunity regulation through direct contact with many immune cells [5,6,30]. Seventy percent of the body’s immune system can be found in the gut-associated lymphoid tissue (GALT)—making it the body’s largest immune organ [31,32]. The GALT is composed of mesenteric lymph nodes (MLNs), Peyer’s patches, and isolated lymphoid follicles (ILFs) [33,34]. Gut-associated immune cells differ in frequency and location along the cross-section of the intestines [32,35,36]. gut lamina propria contains cells from adaptive and innate immune cells, including T-cells and B-cells, natural killer (NK) cells, innate lymphoid cells (ILCs), and dendritic cells (DCs). The intraepithelial lymphocytes (IELs) mainly consist of CD8^+^ T-cells and are found in the intestinal epithelium and are more abundant in the small intestine than the large intestine [29,34,37,38,39]. T helper (Th) 17 and T regulatory cells (Tregs), the most copious T-cells of the gut mucosa, have an inversely related abundance along the intestinal tract. Th17 cells decrease in number from the proximal small intestine to the distal colon, while Tregs are more abundant in the colon [40]. Intestinal T-lymphocytes are the most abundant immune cells in the small and large intestines, distributed thoroughly in the GALT [33]. 

Gut immune cells respond to environmental cues, such as those delivered by the microbiota, and can modulate their response and effector mechanisms accordingly [41,42]. Germ-free (GF) mice show defects in intestinal immune structures, such as diminished amounts and sizes of MLNs and Peyer’s patches [43]. Additionally, GF mice lack fully developed GALTs compared to specific pathogen-free mice [43,44,45]. Gut bacteria also influence the development of naïve CD4^+^ T- cells and can mediate their differentiation into Th1, Th2, Th17, or Tregs, both within—the MLNs—and outside the intestines [46]. Decreased abundance of CD4^+^ T-cells and their lineages are displayed in GF mice compared to isotype-controlled mice [47,48]. 

While it is not completely understood how microbes influence the gut immune system, the most discernible is through the expression of pathogen-associated molecular patterns (PAMPs) that are recognized by pattern recognition receptors (PRRs) on immune cells [49]. PAMP-mediated activation of signaling cascades in PRRs induces the maturation of antigen-presenting cells (APCs), such as DCs and macrophages, and boosts T- and B-cell-related responses. APCs can stimulate the differentiation of Treg and T helper cells, in which the latter stimulates CD8^+^ cytotoxic T lymphocytes—found mainly in the intraepithelial compartment of the gut [28,30,49,50].GF mice display a reduced number and reduced cytotoxicity of intestinal CD8^+^ T-cells, highlighting the importance of gut microbial signals in the proper functioning of cytotoxic cells [51,52]. Furthermore, gut microbiota impacts the ability of gut-associated B-cells, consisting mainly of immunoglobulin (Ig) A-secreting plasma cells, to produce IgA and IgM antibodies [30,53,54]. The abundance and cellular composition of the Peyer’s patches in GF animals are significantly reduced, which in turn diminishes gut IgA levels, emphasizing the critical role of the gut microbiota in maintaining the affinity and functionality of B-cells [51,55,56,57]. Besides the lack of antibody production, GF mice have compromised innate and adaptive immune responses shown by their weaker responses to intracellular and extracellular infections [58,59]. Therefore, the gut microbiota and the immune system are interconnected as proper colonization of the gut ensures that all these immune cells develop and maintain immune balance. 

## 3. Vitamin Absorption and Synthesis by Gut Bacteria 

**Figure 1:**
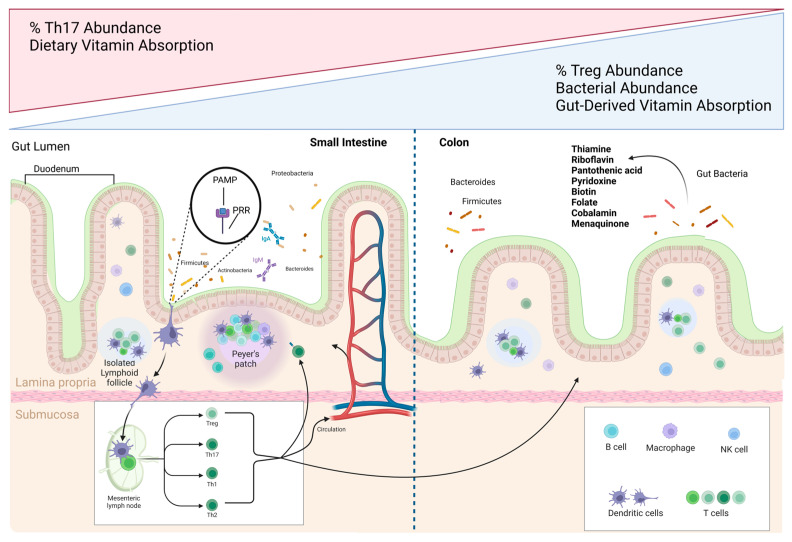
The gastrointestinal (GI) tract contains varying frequencies and distributions of immune cells and vitamin-producing bacteria. The gut-associated lymphoid tissue (GALT) is essential to host immune defense and is comprised of mesenteric lymph nodes (MLNs), isolated lymphoid follicles (ILFs), and Peyer’s patches—found mainly in the small intestine. Gut microbes can express pathogen-associated molecular patterns (PAMPs) and immune cells recognize them through their pattern recognition receptors (PRRs). Antigen-presenting cells (APCs) undergo maturation upon PAMP-PRR interactions on their cell surfaces, which can then lead to the differentiation of naïve CD4^+^ T cells in the MLNs and outside the intestines and boost T- and B-cell-related responses. Th17 and Treg cells are the most abundant T cells in the gut and their concentration gradients along the tract share an inverse relationship. Bacterial abundances also vary along the GI tract, with the duodenum containing the lowest bacterial abundance which increases towards the colon, dominated by Firmicutes and Bacteroides in healthy individuals. Gut-derived vitamins are mainly generated and absorbed in the large intestine, while dietary vitamins are absorbed in the small intestine. Vitamin K2 and most members of the water-soluble B vitamins—thiamine, riboflavin, pantothenic acid, pyridoxine, biotin, folate, and cobalamin—are produced by gut bacteria and play many roles in immune function.

The site of absorption is a critical distinction between vitamins acquired from the diet versus gut bacteria. Most dietary vitamins are absorbed in the small intestine, whereas gut microbial-derived vitamins are predominantly generated and absorbed in the large intestine [39,60]. Some animals, such as rodents, are in fact coprophagic to take advantage of this microbial vitamin production and restriction of this leads to vitamin K and B deficiencies [61,62,63]. Similar to the regional distribution of immune cells, recent reports support an increased bacterial species diversity from the proximal to distal portions of the colon [64,65]. While studies have not yet identified links between regional variation in bacterial diversity and the abundance of immune cell communities, the microbiota is known to play a fundamental role in the induction, development, and function of the innate and adaptive immune system [36,66,67]. As the frequency and distribution of immune cells and bacterial populations vary along the length of the GI tract, the distinct absorption patterns of vitamins may have different implications for the host immune response (Figure 1) [68]. The duodenum contains the lowest bacterial abundance due to the high levels of oxygen, antimicrobial compounds, and lower pH levels from the stomach [68,69,70]. The GI tract includes functionally distinct regions whereby the proximal colon functions primarily in gut fermentation, and the distal colon focuses on extracting fluids and electrolytes [65]. Evidence directly alluding to the effects of these differences on site-specific immune responses in the host remains undetermined.

Bacterial-synthesized vitamins support the growth of the bacteria themselves, and any excess can be released and absorbed to benefit host health. While bacteria require vitamins for their essential cellular functions, not all bacteria are prototrophic and can produce vitamins [71,72]. Vitamin K2 menaquinone and most members of the water-soluble B vitamins—thiamine, riboflavin, pantothenic acid, pyridoxine, biotin, folate, and cobalamin—have been identified as gut microbial-derived vitamins [73,74]. A recent metagenomic study identified that most vitamin-producing microbiota could only synthesize one B or K2 vitamin [75]. In contrast, only 2.7% of those bacteria could produce five or more vitamins [76]. This implies that many prototrophs produce vitamins to support the growth and function of neighboring auxotrophs that cannot produce vitamins. Auxotrophs for vitamins B1, B2, B3, B5, and B9 represent between 20-50% of bacterial communities, whereas auxotrophs for B7 and B12 vitamins are more prevalent, representing up to over 50% of bacterial communities [77]. Out of the eight B vitamins, pyridoxine (B6), folate (B9), and cobalamin (B12) contain the highest estimated percentage of the daily reference intake (DRI) that the gut microbiota could provide at 86, 37, and 31%, respectively. Although bacterial utilization was not considered, the gut microbiota was estimated to produce over a quarter of the dietary recommended intakes for vitamins B6, B9, and B12 [78,79]. While absorption of dietary and gut-derived vitamins is predicted to vary between the small and large intestines, uncertainty remains on how these varied absorption patterns alter the body’s ability to utilize those vitamins and impact their downstream functions [80]. Regardless of these anomalies, these gut-microbial-derived vitamins are critical regulators of intestinal immune homeostasis. Furthermore, gut microbial communities are shown to have an interconnected synthesis and sharing of bioenergetic machinery [81,82]. 

A symbiotic relationship exists between prototrophs and auxotrophs in the gut, where vitamins are cross fed between microbes to support the function and stabilization of gut microbial communities [82]. Bacterial demands for vitamins can differ based on factors that affect the gut microbiome composition, such as age, lifestyle, diet, disease, and alcohol consumption [83,84]. Similarly, host factors can regulate vitamin demands in cells. Increased immune system activation requires enhanced immune cell functions, which may upregulate the need for vitamins to boost this activity [85]. GF mice not only have an underdeveloped immune system, but the absence of vitamin K and vitamin B12-producing bacteria places them at a higher risk of enduring vitamin deficiencies [41,42]. Whether gut bacterial-derived vitamins are sufficient to sustain the host’s immune responses or require additional dietary supplementation remains unclear. However, evidence in the literature emphasizes the importance of vitamins in the gut microbiome in promoting immune cell function and response. 

## 4. Vitamin B6 as a Mediator of Lymphocyte Migration and Responses

Pyridoxine affects the maintenance of the gut barrier by mediating lymphocyte proliferation, activation, and maturation, as well as cytokine and antibody production [86,87]. Specifically, pyridoxine regulates T-cell homeostasis in modulating the Th1/Th2 cell balance, which drives cellular and humoral immunity, respectively [88,89]. Adequate amounts of pyridoxine maintain the Th1 immune response and inhibit Th2-mediated cytokine overactivity. Pyridoxine deficiency negatively impacts both cell-mediated and humoral immunity [89,90]. For example, pyridoxine deficiency decreases CD8^+^ lymphocyte proliferation and activation by suppressing the Th1 response and promoting the Th2 response [89,90]. Downregulation of Th1 responses results in reduced IFN-γ, interference with the induction of delayed-type hypersensitivity (DTH) reactions and lower ability of T-cells to modulate immunosurveillance of invading pathogens [91,92]. Overstimulated Th2 cells excessively produce type 2 cytokines, such as IL-4, IL-5, and IL-13, leading to the overactivity of pro-inflammatory responses [93,94]. This response further decreases antibody response and the formation of the pro-inflammatory cytokines IL1β, IL-2, and IL-2 receptors, which can cause antibody-mediated autoimmune diseases [93,95]. In vivo and in vitro studies have shown that vitamin B6 deficiency leads to defects in thymic epithelial cell function and lowers T lymphocyte abundance, leading to excessive Th2-driven inflammation [89]. Pyridoxine also regulates intestinal immune homeostasis and prevention of excess inflammation by managing IEL migration through sphingosine-1 phosphate (S1P) [96,97]. S1P is a bioactive sphingolipid metabolite and immunoregulator that regulates signaling pathways, such as the NF-κB pathway, in many cell types including macrophages and DCs. As an immunoregulator, S1P generates a gradient through its synthesis and degradation mediated by S1P lyase (SPL). It requires pyridoxal phosphate (PLP), or the biologically active form of vitamin B6, as a cofactor [96,98]. The S1P gradient is crucial in attenuating IEL traffic into the large intestines; however, heightened levels of S1P can lead to augmented inflammation by dysregulation of its maintenance of vascular integrity [99]. Vitamin B6 supplementation was shown to promote anti-inflammatory properties by promoting SPL, thus reducing S1P expression [98,100]. Therefore, vitamin B6 is essential in intestinal immunosurveillance by ensuring proper lymphocyte balance and IEL migration and activation. 

## 5. Vitamin B9 in Treg Immunosurveillance 

**Figure 2:**
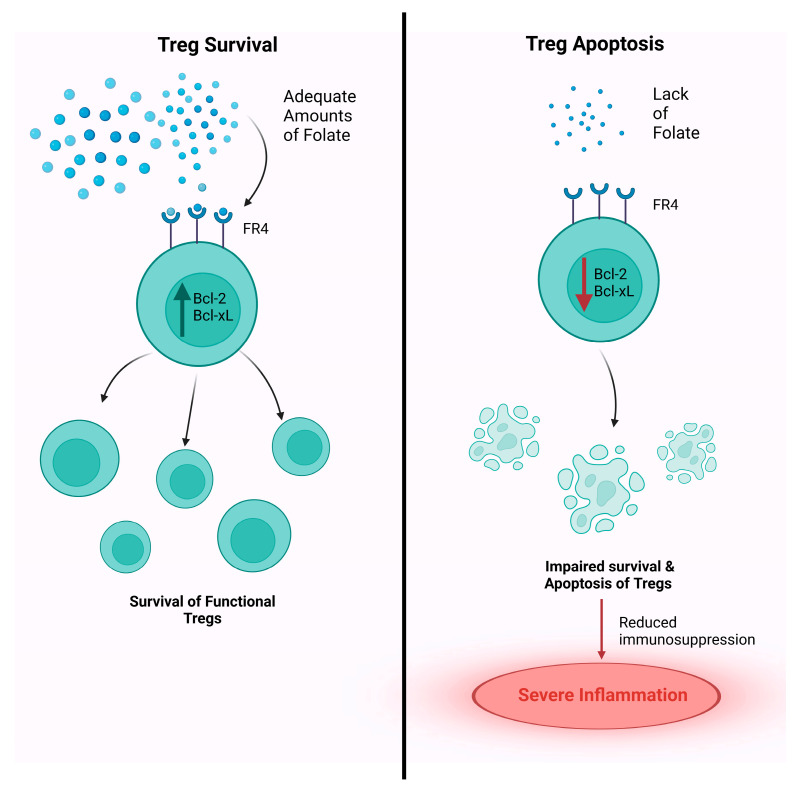
Folate is required for the survival of mature Tregs. Once naïve T cells differentiate into Tregs, they highly express folate receptor 4 (FR4), which regulates the development and function of Tregs. The binding of folate to FR4 stimulates the production of the anti-apoptotic molecules Bcl-2 and bcl-xL, allowing for the survival of functional Tregs. Folate deficiency affects the survival of Tregs, leading to a reduction Bcl-2 and Bcl-xL and hence, an increase in Treg apoptosis. Impaired Treg function may impair immunosuppression and lead to increased susceptibility to intestinal inflammation.

Folate’s main contribution to immune homeostasis derives from its requirement in Treg function [101]. Folate is required for immunosurveillance by stabilizing Tregs, specifically peripheral Tregs (pTregs), and aiding T-cells traffic from the colon to distant sites to assert their immune response [102,103]. Folate is required for the survival of mature Tregs but not the differentiation of immature T-cells into mature Tregs [103]. In vitro culture of Tregs under low vitamin B9 conditions leads to impaired cell survival, with decreased expression of anti-apoptotic Bcl2 molecules [104]. However, naïve T-cells retain their ability to differentiate into Tregs; which suggests that vitamin B9 is a survival factor for Tregs [103]. Once naïve T-cells differentiate into Tregs, they highly express folate receptor 4 (FR4), which regulates the development and function of Tregs [101,105]. High expression of FR4 on the surfaces of Tregs marks the important role of folate in the survival of these cells (Figure 2) [106,107]. Studies emphasized folate’s effect on the survival of Tregs by blocking FR4, which led to a significant reduction in the anti-apoptotic molecules Bcl-2 and Bcl-xL and hence, an increase in Treg apoptosis [107,108,109]. Mice fed with a vitamin B9-deficient diet exhibit increased susceptibility to intestinal inflammation [108]. Consistent with these findings, a deficiency of dietary vitamin B9 results in the impaired survival of the Tregs population in the small intestine [103]. Moreover, certain conventional T-cells that become autoreactive undergo apoptosis during development; however, a subset of cells undergoes a state of anergy known as T_an_ [110]. Gut microbial-derived folate can induce the conversion of conventional T-cells into T_an_ [103,106]. T_an_ also possesses FR4, indicating an overlapping relationship between T_an_ and Tregs [111]. T_an_ contains a greater number of methylation sites in the periphery compared to the thymus, which promotes transcriptional silencing and gene inactivation. The instability promoted by these methylation sites in T_an_ can promote the interconversion between T_an_ and Tregs [112,113,114]. Therefore, gut microbial-derived folate can drive epigenetic modifications through the loss of methylation sites in T_an_ to induce Treg replenishment. Decreased microbial-derived folate has been related to dysbiosis and the proliferation of autoreactive T lymphocytes that can lead to autoimmunity [112,115,116,117]. Reduced folate can lead to reduced SCFA production and cause an increased abundance of autoreactive immunogenic T effector cells (T_eff_) while reducing the abundance of Tregs [112,118,119,120]. Decreased Tregs trafficking from the colon to distant sites shifts the opportunity for such movement to T_effs_ [117,118]. T_effs_ target autoimmune target sites and skew the T_eff_ to Treg ratio to override immune privilege and trigger autoimmune diseases such as colitis [117,121,122,123]. The effects of folate-deficiency on Treg immune profiles and gut dysbiosis can be consequential in immunotherapies such as ICIs, specifically in patients suffering from autoimmune diseases. For instance, patients with colitis may be characterized with dysbiosis in the gut ecosystem that potentiates the malabsorption of folate. Furthermore, ICIs can trigger inflammation throughout the body, particularly in organs that are targeted in autoimmunity, such as the intestines, and cause severe inflammation in those patients by dysregulating the Treg and T_eff_ ratio. These adverse effects (AEs) may be detrimental to cancer patients suffering from such autoimmune diseases and folate deficiency, as the ICIs can enhance the AEs and may cause severe complications in patients [115,123,124]. Therefore, adequate folate is required in the surveillance and the immunological network of Tregs, which are linked to the control of autoimmunity and gut dysbiotic changes and should be taken into consideration in future immunotherapy studies.

## 6. Vitamin B9 and B12 in Their Role as Co-Dependent Immunomodulators 

**Figure 3:**
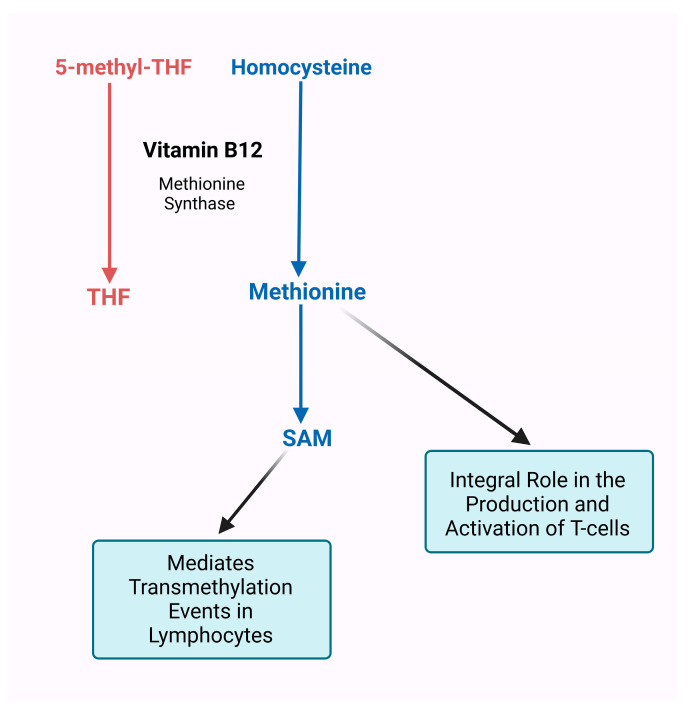
Vitamin B12 and 5-methyl-tetrahydrofolate (THF) interact to produce methionine for T cell activation. Methionine synthase is a vitamin B12-dependent enzyme that produces methionine via homocysteine and 5-methyl-THF. The uptake of methionine is required in T-cells to provide methyl groups to RNA and DNA, needed for the cells to proliferate and differentiate. The absence of methionine leads to the failure of T-cell expansion and a weakened cell-mediated immune response. Methionine is essential to synthesize S-adenosylmethionine (SAM), which plays a major role in transmethylation reactions that support immune function. Lymphocytes depend upon SAM in activated states for transmethylation events involving DNA, RNA, or protein molecules, which are required for maintaining cell function.

Interestingly, dietary and microbial-derived folate requires cobalamin (vitamin B12) to be absorbed in the intestinal wall, as without it, folate remains trapped in the colon [125]. Vitamin B12 is a cofactor for methionine synthase—important for catalyzing the formation of methionine from homocysteine—which is required to demethylate the inactive 5-methyl tetrahydrofolate to its active form tetrahydrofolate (THF) [126]. In cobalamin deficiency conditions, 5-methyl THF augmentation evokes a secondary folate deficiency affecting protein and DNA production as well as altering immune cell regulation and defense [85,127]. An inadequate amount of cobalamin can negatively affect folate functions, thereby inducing cobalamin deficiency that consequently promotes folate deficiency [128,129]. Upon absorption, cobalamin requires 5-methyl THF to produce methionine from homocysteine [130,131]. T-cells import methionine for protein synthesis and provide methyl groups to methylate the RNA and DNA required to drive the proliferation and differentiation of T-cells. Upregulation of the methionine transport Slc7a5 requires T-cell activation and is the rate-limiting factor for the generation of methyl groups [131,132]. In the absence of methionine, T-cells fail to expand and develop, leading to a weakened T-cell-mediated immune response [131,133]. Therefore, methionine is an integral portion of the production and activation of T-cells. Methionine is essential in supporting biochemical reactions by working alongside cobalamin and folate to synthesize S-adenosylmethionine (SAM) [130,134]. SAM is a major methyl donor in transmethylation reactions and is important for modulating biochemical reactions that support immune function [135,136,137,138,139,140,141]. T-cell activation upregulates methyltransferases, which depend upon SAM to methylate DNA, RNA, or proteins to allow for the differentiation and proliferation of those T-cells. The amount of SAM consumed is 4-10 times higher in activated lymphocytes compared to resting lymphocytes, and even in resting lymphocytes, the rates are 3–5 times greater than other cell types [142,143]. These findings indicate that lymphocytes are more sensitive to transmethylation events mediated by SAM for their function and activation than other cell types. Therefore, the interconnection of vitamin B12 with vitamin B9 through methionine and SAM metabolism demonstrates the necessity of not only ensuring that vitamin B12 can perform its essential functions but also ensuring that folate can be absorbed and perform its downstream immunomodulatory operations (Figure 3). Furthermore, folate affects cell-mediated immunity and immune cell proliferation [144,145]. Folate deficiency leads to reduced T-cell blastogenic response to certain mitogens [103,146]. Studies have also shown that antibody response is affected in folate-deficient hosts [147,148,149]. These alterations to humoral and cell-mediated immunity can reduce infection resistance [150,151].

## 7. Vitamin B6, B9, and B12 in the Regulation of Cytotoxic Immunity 

Pyridoxine, folate, and cobalamin are involved in maintaining and enhancing cytotoxic and killer cells [152]. In vitro and in vivo studies show that the metabolism of these vitamins is required for CD8^+^ T-cell proliferation and their effector functions [118,127,146,153,154,155,156]. In contrast, a deficiency of these vitamins promotes DNA damage by arresting cell division at the S-phase and reduces cytotoxic cell expansion [153]. Furthermore, this deficiency inhibits cytotoxic T and NK cell activity, increasing dysregulation of the gut barrier and thus heightening the onset of infections [77]. Vitamin B9 deficiency has been suggested to inhibit CD8^+^ T-cell regulation as the ratio of CD4^+^ to CD8^+^ lymphocytes increases [146,157,158]. Pyridoxine deficiency leads to decreased NK cell activity and the increased onset of infection [154,159]. Similarly, cobalamin and folate regulate CD8^+^ T- and NK cell proliferation and antibody and Ig production in B-cells [13,158]. Reductions in Ig production such as reduced IgG and IgM levels, result in host immunodeficiency, putting the host at a higher risk of immune-prone diseases [55,160]. Similarly, reduced CD8^+^ T-cell and NK cell cytotoxicity can increase the host’s risk of infection and chronic inflammation by altering their immune risk phenotype [161,162,163]. Cytotoxic CD8^+^ T-cells constitute an important player in ICIs as they are associated with enhanced ICI-efficacy in cancer patients and hence, prolonged survival [164]. A lack of T-cells, specifically within the tumor, can lead to primary resistance to immunotherapy and poor disease prognosis in patients. Hence, vitamins B6, B9, and B12 are crucial in deciphering the efficacy of immunotherapy through their ability to regulate cytotoxic immunity and immune cell infiltration in tumors [165].

## 8. Vitamin K2 and D: More Than Calcium Metabolism and Bone Health 

Vitamin D and vitamin K are fat-soluble vitamins that play an essential role in calcium metabolism and bone health. Vitamin D, or calcitriol, promotes the production of vitamin K-dependent proteins for calcium absorption, while vitamin K works to activate those proteins [166,167]. Evidence supports these vitamins work together not only in bone health but also in immune health [166,168]. Vitamin K can be separated into two distinct types, vitamin K1 and vitamin K2, each playing varying but significant roles. Vitamin K1 (phylloquinone) is derived from green plants and is acquired through the diet [169,170]. Vitamin K2, or menaquinone (MK), presents in several forms, predominating from obligate and facultative anaerobes [171]. Like vitamin B12, MKs can be remodeled by gut bacteria, hinting at possible modulation of the gut microbiome composition by the vitamin [172,173]. Gut microbiota with abundant levels of diverse bacteria can provide adequate levels of MK to significantly impact the host, pointing to an association between gut microbiota and sufficient vitamin K levels [172]. Similarly, vitamin D can be sourced from the diet as vitamin D2 or D3, or from the skin—through a photolytic process—as vitamin D3. Vitamin D2 or D3 is then converted into the inactive prohormone 25-hydroxycholecalciferol (25D) in the liver and then hydroxylated by the CYP27B1 enzyme into its active form 1,25-dihydroxyvitamin D (1,25D) [174]. While it is noted that vitamin D is not made in large quantities by gut bacteria; both 1,25D and 25D may possess bi-directional relationships with the gut microbiome and the immune system [175]. Vitamin D supplementation has been suggested to elevate the abundance of probiotics and to specifically increase bacteria that produce the SCFA butyrate, such as Akkermansia and Bifidobacterium [176]. Butyrate has been extensively reviewed for its ability to enhance the immune system-gut connection by modulating immune homeostasis and promoting healthy inflammatory responses in the colon [13,177,178,179,180]. Interestingly, evidence suggests that individuals with higher vitamin D levels possess a greater abundance of butyrate-producing bacteria shown to be associated with gut microbial diversity and better overall gut-immune health [181]. Furthermore, circulating levels of vitamin D may be involved in regulating immune homeostasis, which is in part tied to its interactions and alterations to gut microbiota composition [182]. Therefore, both MKs and Vitamin D levels demonstrate an intertwined relationship between gut microbiota composition and immune response regulation.

### 8.1. Vitamin K2 and D: Players in Effector and Tolerogenic Immunity 

**Figure 4:**
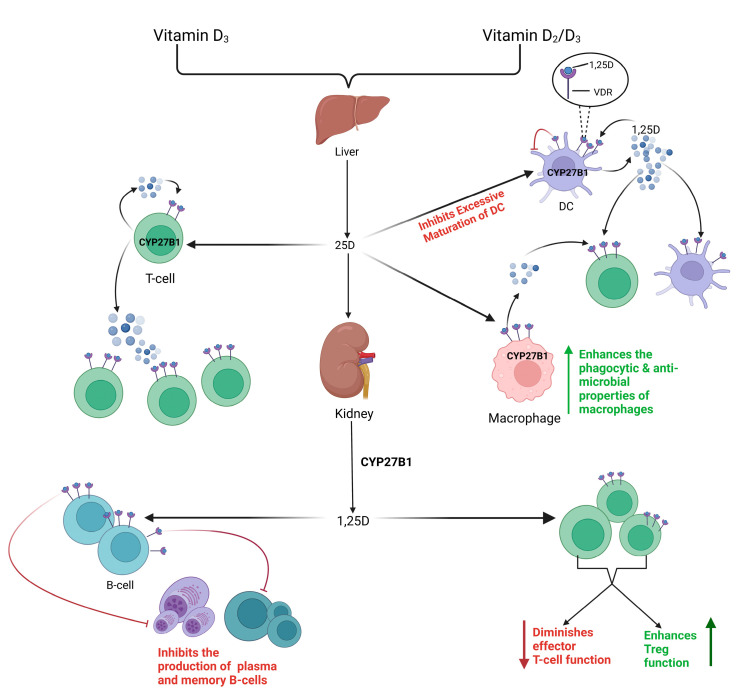
Vitamin D receptors (VDR) and CYP27B1 are expressed in immune cells and regulate immune responses. The presence of CYP27B1 in antigen-presenting cells (APCs), T cells, B cells, and monocytes allows these immune cells to produce 1,25D, which can bind to its receptor VDR. The 1,25D-VDR complex inhibits the excessive maturation of dendritic cells (DCs). In monocytes and macrophages, 1,25D boosts their phagocytic capabilities and antimicrobial effects. 1,25D contribute to B-cell homeostasis by directly inhibiting memory and plasma cell generation. Furthermore, different forms of vitamin D can affect the proliferation and differentiation of T cells, such as the intracrine conversion of 25D to 1,25D by T cells. T cells may also be affected downstream of the production of 1,25D in APCs and macrophages. 1,25D can elevate the levels of circulating Tregs and reduce the effector functions of cytotoxic and helper T cells, which supports tolerance and highlights its anti-inflammatory properties.

The combined effect of vitamin D and vitamin K2 in anti-tumor responses may be due to synergistic improvement in cellular differentiation outcomes and reduced hypercalcemic complications of standalone supplementation [183]. Potential reasoning for these effects may stem from calcitriol and MK’s comparable anti-inflammatory and immune-regulatory properties [184,185]. Interestingly, vitamin D receptors (VDR) and CYP27B1 are expressed in many cells, including immune cells such as APCs, T-cells, B-cells, and monocytes (Figure 4) [186,187,188]. The discovery of the extra-renal synthesis of 1,25D by immune cells suggests a more widespread influence of vitamin D on the immune system than ever before. While vitamin D is known to boost the phagocytic capabilities of monocytes and macrophages, it can promote their antimicrobial activity through VDR acting as a gene transcription regulator. Furthermore, 1,25D is important for tolerogenic modulation. The presence of CYP27B1 in DCs allows for the generation of 1,25D concentrations and modulation of immune responses [189,190]. Furthermore, the 1,25D-VDR complex in DCs inhibits the excessive maturation of these cells [191,192]. In vivo studies of VDR-KO mice found high levels of mature DCs and abnormal DC chemotaxis, highlighting the importance of vitamin D-VDR in self-tolerance development and autoimmunity [193,194]. Furthermore, vitamin D may possess immunological properties in the adaptive immune system via the expression of VDR on both T- and B-cells [195,196]. Direct effects of 1,25D on B-cell homeostasis are highlighted through its ability to inhibit memory and plasma cell generation, and due to its pro-apoptotic properties in autoreactive antibodies, which provide clinical significance in autoimmune diseases [174,195,197]. Furthermore, the different forms of vitamin D influence the proliferation and differentiation of T-cells via direct and indirect mechanisms. Direct impacts include the endocrine effects of systemic calcitriol on T-cells, the intracrine conversion of 25D to 1,25D by T-cells, and the paracrine effect of 1,25D stemming from DCs. T-cells may also be affected indirectly through the important role of 1,25D in APCs [198]. Vitamin D has been shown to attenuate Tregs phenotypes by elevating the levels of circulating Tregs and diminishing the effector functions of cytotoxic and helper T-cells to promote anti-inflammatory responses and support tolerance [19,197,199,200,201]. Therefore, the relationship between 1,25D and VDR is crucial for regulation of the innate immune system in promoting antimicrobial and tolerogenic immune responses, as well as in regulating adaptive immunity [202,203].

### 8.2. Vitamin D and K Deficiencies as Preventable Risk Factors 

Vitamin D’s role in immune responses highlights how vitamin D deficiency may skew these immune responses and become a risk factor for autoimmune diseases such as ulcerative colitis, rheumatoid arthritis, and thyroid diseases [204]. These risk factors should be considered in the context of vitamin D levels and ICI administration in cancer patients. ICIs have several AEs that correspond with the depicted autoimmune diseases related to vitamin D deficiency, targeting organs such as the thyroid, the bones, and the intestines. As vitamin D is a protective factor for several autoimmune diseases, it may be able to potentiate these ICI-induced AE by regulating immune responses [204,205]. 

The crucial role of vitamin D in the balance between effector and tolerogenic immunity can be demonstrated by vitamin K2 in relation to bone health. At in vitro doses related to bone cell function, inhibition of T-cell proliferation was suggested as not vitamin K1-induced but rather vitamin K2-specific, demonstrating vitamin K2’s immunomodulatory properties in T lymphocytes [206]. Further research is needed to better understand the immunoregulatory function of vitamin K2 on T-cells, specifically in immune cells.

Moreover, vitamin D is a modifiable risk factor for haematological diseases, as lower levels of vitamin D in patients with these diseases correlate with worse disease outcomes [207]. Vitamin D levels are low in patients with acute leukemia and these levels are shown to decline further after remission induction therapy [208]. Conversely, in Hodgkin’s lymphoma, supplemental vitamin D reduces the rate of tumor growth, demonstrated by the improved chemosensitivity of the tumors compared to vitamin D or chemotherapy treatments in standalone [207]. Similarly, higher levels of vitamin K are associated with anti-cancer effects, as individuals with a higher intake of vitamin K from their diet had a reduced risk of developing non-Hodgkin’s lymphoma [209]. Furthermore, vitamin K2 has been shown to prevent lymphoma in Drosophila [210]. Therefore, the serum levels of vitamins may not only be of use in differentiating the risks of developing various types of haematological disorders, but their role in immune responses may also provide downstream applications in cancer therapies. 

### 8.3. Vitamin K2 and D in the Expression of Pro-Inflammatory Markers 

Vitamin K2 and Vitamin D have anti-inflammatory effects marked by their ability to affect the expression of inflammatory markers [185,211,212,213]. Vitamin K2 has been shown to prevent cytokine storm—a phenomenon in which stimuli lead to extreme inflammatory response through rapid secretion of inflammatory cytokines [214,215]. Specifically, MK4 reduced the expression of NF-κB, a transcription factor for IL-6, which can reduce this cytokine levels [216,217]. MK4 is suggested as a more potent anti-inflammatory compound than vitamin K1 in primary fibroblastic and monocytic cell lines [213]. COVID-19 patients supplemented with vitamin K2 had reductions in IL-6 and tumor necrosis factor-alpha (TNFα) expression [215]. Reports to date have shown that lower levels of MK are associated with a more severe COVID-19 disease prognosis, which may correspond to the increased prevalence of IL-6 secretion and cytokine storms in those patients [218,219]. Similarly, vitamin K deficiencies are marked in many chronic GI inflammatory disorders and MK4 supplementation in a murine model of colitis was shown to provide immunosuppressive benefits by reducing pro-inflammatory cytokine production such as IL-6 [220]. Additionally, an in vitro atopic dermatitis study found that vitamin K2 significantly inhibited levels of the pro-inflammatory cytokines IL-17 and TNF-α [221]. Similarly, vitamin D’s importance to tolerogenic response extends to its ability to decrease the expression levels of T-cell cytokines, through VDR’s ability to suppress transcription of cytokine genes in activated T-cells [222,223,224]. Furthermore, evidence suggests that vitamin D can alter the Th1/Th17 pro-inflammatory phenotype to a more anti-inflammatory Th2/Treg phenotype [202,225]. Treatment with calcitriol has been credited to the inhibition of pro-inflammatory Th1 cytokines, specifically IL-1, IL-6, IL-8, IL-12, and TNF-α, as well as the induction of anti-inflammatory Th2 cytokines such as IL-10 and IL-4 [226,227,228,229,230,231,232,233,234,235,236]. A study found significant suppression of the pro-inflammatory IL-6 in healthy adults supplemented with a high dose of calcitriol [237]. Similar to MK, low vitamin D levels are associated with increased inflammatory markers, such as IL-6, in COVID-19 patients at risk of developing severe inflammatory conditions and higher mortality rates [238,239]. Furthermore, Vitamin D inhibits Th17 activity by suppressing IL-17 production at the transcriptional level in human T-cells, revealing a shift towards a tolerogenic phenotype [40,174]. Therefore, both vitamin K2 and D possess anti-inflammatory properties credited to their ability to regulate pro-inflammatory markers. 

## 9. Vitamins and Immunotherapy: Enhancement of Therapy and Prevention of Side Effects 

Gut microbial-derived vitamins have immunomodulatory properties and may enhance ICI response rates [84,176]. The capability of the host’s immune system to fight against tumor cells is key to successful outcomes in immunotherapy. Unfortunately, ICI immunotherapies are shown to be only clinically effective in 10-40% of cancer patients and can trigger autoreactive adverse reactions [240,241]. Novel treatment strategies that target gut microbiota composition and diversity are currently in the works to overcome such limitations [242,243,244,245,246]. 

Since 2015, several preclinical and clinical studies have been hailed as milestones in the link between gut microbiota and ICI responses. Notably, certain bacterial species (i.e., Bifidobacterium, Akkermansia) were marked as elevated in immunotherapy responders versus non-responders throughout multiple studies [247,248]. Oral administration of Bifidobacterium spp. in tumor-bearing mice promoted DC maturation leading to enhanced CD8^+^ T-cell priming and improved anti-PD1 efficacy [247,249,250]. Furthermore, supplementation with *Bacteroides fragilis* in antibiotic-treated mice augmented the efficacy of anti-cytotoxic -T-lymphocyte- associated protein 4 (CTLA-4) therapy by stimulating DC maturation and thus, Th1 immune-mediated responses [248]. Similarly, enhanced anti-tumor immunosurveillance to immunotherapy and elevations in intestinal metabolites were found with oral administration of *Akkermansia muciniphila*—in which several strains are proposed to be vitamin B12 producers—in animal studies [251,252]. In human studies, fecal microbiota transplantation (FMT) from ICI responders to antibiotic-treated mice ameliorated anti-tumor responses, which was not seen in FMTs from non-responders [253,254]. Oral supplementation with *A. muciniphila* following FMT in non-responders improved anti-PD1 response in lung, renal cell, and urothelial carcinoma patients by recruiting CCR9^+^ CXCR3^+^ CD4^+^ T-cells into tumor beds [254,255]. In melanoma patients, ICI responders with higher abundances of Faecalibacterium and Ruminococcaceae—auxotrophic for most B vitamins—showed increased CD4^+^ T- and CD8^+^ T-cells in their periphery [72,253,256,257]. In 2021, two seminal clinical trials displayed that FMT from ICI responders with anti-PD1 therapy reprogrammed the tumor microenvironment to overcome resistance to PD1-blockade in a subset of melanoma patients [258,259]. Similarly, an emphasis on the relationship between gut microbiota and immunotherapy response efficacy has been implicated in studies looking at the link between antibiotic-induced dysbiosis and attenuations to ICI efficacy [15,260,261,262,263,264]. Antibiotic-treated patients with advanced solid tumors displayed reduced survival rates and poor ICI responses, which were associated with reduced gut microbiota diversity and abundance [56,263,265]. Furthermore, dysbiotic gut microbiomes may be unable to perform vital functions which may impact vitamin biosynthesis and ultimately deplete vitamin-producing bacteria [266,267,268]. Ampicillin, Chloramphenicol, Ciprofloxacin, and Vancomycin have been shown to reduce vitamin biosynthesis by affecting the gut transcriptome [266]. Therefore, the interconnection between the gut microbiota and the immune system has the potential to shape the response to immunotherapy.

**Figure 5:**
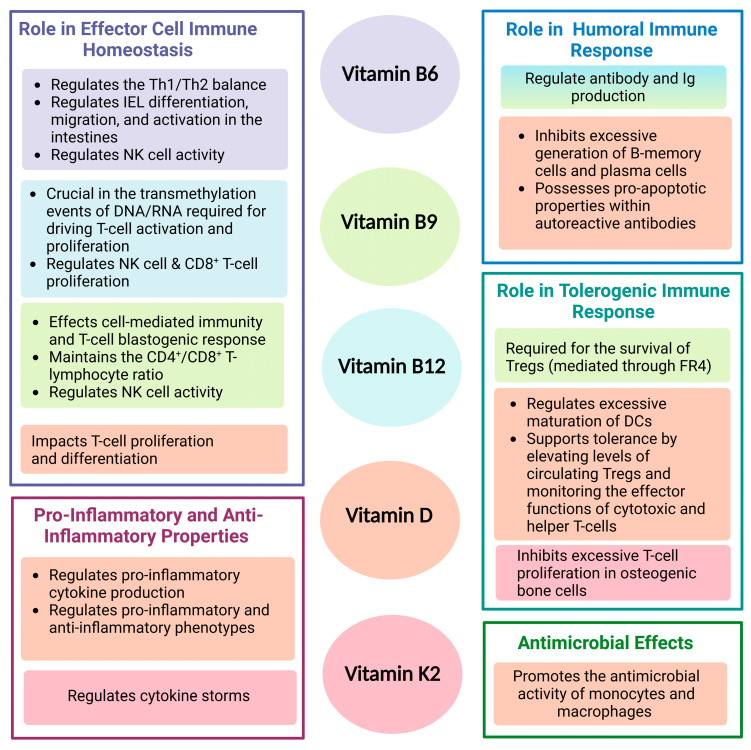
Summary of the Immune Functions of Vitamins B6, B9, B12, D, and K2.

A symbiotic gut microbiota provides adequate endogenous vitamin production to support immune function (Figure 5), and inversely, vitamins can beneficially modulate the ecology of the gut microbiota. Disruptions to the bidirectional relationship between vitamins and the gut microbiome can potentially cause the loss of beneficial bacteria and vital micronutrients [84]. It is currently unfeasible to determine the exact proportion of microbially-derived vitamin deficiency compared to dietary vitamin deficiency in individuals. However, vitamin deficiency has been postulated as a link to gut dysbiosis—marked by a detrimental loss in probiotics and commensals—causing detrimental effects on the immune system and potentiating inflammatory diseases [176,269,270]. B vitamins, vitamin K, and many dietary vitamins have established immunoregulatory properties that coincide with positive immunotherapy response rates. The mechanistic role of vitamin B6 in inducing T-cell subtype determination and inducing PD-1/PD-L1 blockage renders it a natural immunoregulator and a potential option for combination immunotherapy [271]. Comparably, PD-L1 expression is significantly reduced in the presence of vitamins B6 and B9 and thus, corresponded to reduced proliferation of pro-monocytic lymphoma cells in vitro [127]. Furthermore, Vitamin D supplementation can suppress tumor angiogenesis and induce anti-inflammatory effects within the tumor microenvironment, promoting apoptosis and autophagic death of tumor cells [272]. In a clinical trial of tuberculosis patients, supplementation with high doses of vitamin D and adjunctive therapy reduced inflammatory responses more quickly than in patients who received no supplementation [273]. These results support vitamin D’s tolerogenic immunologic properties that may provide potential therapeutic promises as an adjuvant with cancer therapies [190]. Vitamin D has also shown potent anti-cancer benefits alongside treatment in patients with melanoma, prostate, breast, and colorectal cancers [24]. Similarly, vitamins may modulate the human gut microbiome and immune barrier function in terms of metabolic activity and bacterial composition. Evidence suggests that vitamin D and B vitamins increase the abundance of butyrate-producing bacteria and the gut-mucosal integrity bacteria *A. muciniphila* [74]. The effect of vitamins in sustaining and promoting the growth of such immunomodulatory bacteria emphasizes the bidirectional symbiosis between vitamins and gut bacteria and its potential for immunotherapy enhancement. Prospective studies are needed to analyze alterations in metabolite production from gut microbial modulation production and the indirect impacts of their vitamins on immunotherapy response. 

There are many immune-related adverse events (AEs) that occur in ICI treatments, such as colitis and peripheral neuropathy [274,275,276,277,278]. Several bacterial species, including *Bacteroidaceae*, *Bifidobacterium animalis*, *Bifidobacteria infantis*, *Faecalibacterium prausnitzii*, and *Lactobacillaceae* are associated with the maintenance of tolerogenic responses in the gut [51,264,279,280,281,282,283,284,285,286,287,288,289]. The bacteria are deemed protective against ICI-induced colitis through nitric oxide production, shifts in the Th1/Th2 balance, and induced Treg differentiation. These protective effects may be potentiated by the synthesis of group B vitamins by these bacterial species [24,28,30,261,290,291,292,293,294]. Therefore, vitamins, or specifically gut-derived vitamins, may be critical in the interplay between the gut microbiota and the prevention of ICI-induced AEs. Clinical evidence suggests that vitamin B12 can efficiently reduce the toxicity of cancer treatments due to its necessity in the nervous system and red blood cell synthesis [130,295]. Vitamin B12 is used alongside several chemotherapies to hinder the severity of drug-induced peripheral neuropathy [127,296]. Similarly, patients treated with ICIs while being administered vitamin D reported a reduced risk of ICI-induced colitis [205]. It remains unclear the extent to which these immune responses vary between microbial-derived or dietary vitamins and if these differences could drive variations in immunotherapy sensitivity in patients. Nevertheless, vitamins have the potential to enhance immunotherapy responses while simultaneously preventing AEs, however, clinical trials are needed to determine the specific roles of vitamins and how their levels correlate to responses in the immune-gut axis of cancer patients. 

## 10. Conclusions 

The gut microbiome has gained immense attention over the past decade, due to the interconnection of its microbes and products with the immune system and its potential as an immunomodulatory target in anti-cancer therapy. The extent of the fundamental mechanisms and pathways of gut-microbial vitamins in immune-mediated cancers remains elusive; however, the bidirectional relationship between vitamins and the gut-immune axis warrants potential consideration for immunotherapy. Gut-derived vitamins are essential in ensuring cellular and humoral immunity’s proper development and function, playing an integral defensive role in anti-tumor responses. These micronutrients, in turn, drive a healthy and diverse gut microbial composition that supports the immune system. Such effects shed light on the potential to improve clinical outcomes in immunotherapy patients and minimize the risk of adverse-therapeutic effects. As many questions about gut-derived vitamins remain unanswered, many more studies, including randomized clinical trials, are required to elucidate their role in the efficacy of immunotherapies and their specific effects on immune cells and anti-tumor immune responses.
